# Mitochondrial and Y-chromosome diversity of the Tharus (Nepal): a reservoir of genetic variation

**DOI:** 10.1186/1471-2148-9-154

**Published:** 2009-07-02

**Authors:** Simona Fornarino, Maria Pala, Vincenza Battaglia, Ramona Maranta, Alessandro Achilli, Guido Modiano, Antonio Torroni, Ornella Semino, Silvana A Santachiara-Benerecetti

**Affiliations:** 1 Dipartimento di Genetica e Microbiologia, Università di Pavia, 27100 Pavia, Italy; 2 Dipartimento di Biologia Cellulare e Ambientale, Università di Perugia, 06123 Perugia, Italy; 3 Dipartimento di Biologia, Università di Roma 'Tor Vergata', 00173 Roma, Italy; 4 Current address: Human Evolutionary Genetics, CNRS URA 3012, Institut Pasteur, Paris, France

## Abstract

**Background:**

Central Asia and the Indian subcontinent represent an area considered as a source and a reservoir for human genetic diversity, with many markers taking root here, most of which are the ancestral state of eastern and western haplogroups, while others are local. Between these two regions, Terai (Nepal) is a pivotal passageway allowing, in different times, multiple population interactions, although because of its highly malarial environment, it was scarcely inhabited until a few decades ago, when malaria was eradicated. One of the oldest and the largest indigenous people of Terai is represented by the malaria resistant Tharus, whose gene pool could still retain traces of ancient complex interactions. Until now, however, investigations on their genetic structure have been scarce mainly identifying East Asian signatures.

**Results:**

High-resolution analyses of mitochondrial-DNA (including 34 complete sequences) and Y-chromosome (67 SNPs and 12 STRs) variations carried out in 173 Tharus (two groups from Central and one from Eastern Terai), and 104 Indians (Hindus from Terai and New Delhi and tribals from Andhra Pradesh) allowed the identification of three principal components: East Asian, West Eurasian and Indian, the last including both local and inter-regional sub-components, at least for the Y chromosome.

**Conclusion:**

Although remarkable quantitative and qualitative differences appear among the various population groups and also between sexes within the same group, many mitochondrial-DNA and Y-chromosome lineages are shared or derived from ancient Indian haplogroups, thus revealing a deep shared ancestry between Tharus and Indians. Interestingly, the local Y-chromosome Indian component observed in the Andhra-Pradesh tribals is present in all Tharu groups, whereas the inter-regional component strongly prevails in the two Hindu samples and other Nepalese populations.

The complete sequencing of mtDNAs from unresolved haplogroups also provided informative markers that greatly improved the mtDNA phylogeny and allowed the identification of ancient relationships between Tharus and Malaysia, the Andaman Islands and Japan as well as between India and North and East Africa. Overall, this study gives a paradigmatic example of the importance of genetic isolates in revealing variants not easily detectable in the general population.

## Background

**Figure 1:**
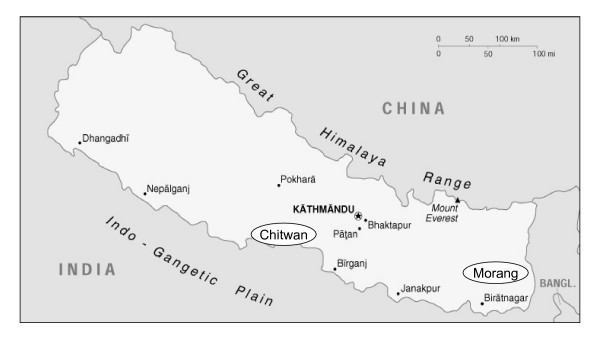
**Geographic map of Nepal**. Sampled areas, in circles.

Terai, a highly malarial region of South Nepal bordering on India (Figure 1), was until a few decades ago, when malaria was eradicated, inhabited almost exclusively by Tharus, one of the oldest and the largest indigenous people of Terai. This group is known for their resistance to malaria as evidenced by their decreased malarial morbidity compared to sympatric Nepalese populations [1], a phenomenon not completely clarified at the genetic level. It was only after substantially full malaria eradication, through a program for malaria control started in 1956, that several other Nepalese populations migrated and settled in Terai. Tharus live throughout the length of the country (mainly in the northern strip of Terai) in villages very close to, or even inside, the previously malarial forested zones. Although culturally and linguistically very heterogeneous, they consider themselves as a unique tribal entity subdivided into three main groups (western, central and eastern).

Because of its geographic position in a boundary area of Central Asia, Terai was a preferential passageway during the dispersal of many prehistoric and historic populations, thus Tharus might have retained genetic traces of ancient migratory events. Until 1980, however, their genetic structure was almost unknown and, on the basis of some classical serum markers [2] and physical features [3], they were considered a 'Mongoloid' tribe. Subsequent studies, carried out on mitochondrial DNA (mtDNA) RFLPs, however, provided further support for the presence of a Tharu East Asian component [4-8] and showed other genetic characteristics of unclear origin [9]. In addition, heterogeneity among the three groups was also evidenced [5,9] by the different distribution of the malarial related α-thal gene [10].

Even in more recent phylogeographic studies encompassing a large number of populations and including Tharu samples, mostly from Uttar Pradesh [11-16], the Tharu genetic structure was not completely clarified.

The present availability of more advanced techniques, which allow molecular analyses at a much higher level of resolution with extremely small amounts of DNA, prompted us to once again address the issue of the genetic origin of the Tharus, by analyzing both their mtDNA (including sequencing of entire mtDNAs) and Y-chromosome (SNPs and STRs) variation.

## Methods

### The sample

The sample consisted of 173 Tharu DNAs from male blood specimens collected more than 25 years ago, soon after the massive immigrations of other populations into Terai following malaria eradication, and 104 Indians. The Tharu sample was composed of three groups from different villages: two in the Chitwan district of Central Terai (Th-CI and Th-CII) and one in the Morang district of Eastern Terai (Th-E) (Figure 1). The Indian sample also was composed of three groups: Hindus from Terai (H-Te, collected in the Chitwan district), Hindus from New Delhi (H-ND) and tribals from Andhra Pradesh (T-AP). Absence of close relationships between the individuals was ascertained through interview data. When necessary, genomic amplification of DNA was performed by using the Amersham GenomiPhi kit.

This research has been approved by the Ethic Committee for Clinical Experimentation of the University of Pavia, after having verified the conformity to the international rules.

### MtDNA analyses

Affiliation within mtDNA haplogroups was first inferred through the sequencing of a region ranging from 630–876 base pairs (bps) from the control region that, according to the rCRS [17], encompasses the entire hypervariable segment I (HVS-I) and part of HVS-II, then confirmed through a hierarchical survey by PCR-RFLP/DHPLC/sequencing of haplogroup diagnostic markers in the coding regions [see Additional file 1]. The 9-bp deletion/insertion polymorphism, already studied in a subset of these populations [6], was also evaluated in all samples.

**Figure 2:**
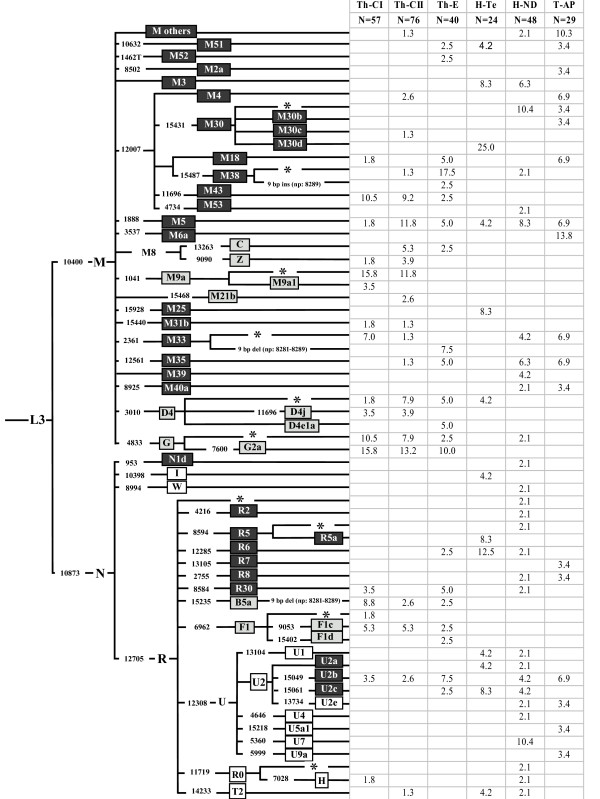
**Phylogeny and frequencies (%) of mtDNA haplogroups in the populations studied**. Haplogroups (East Asian in grey; West Eurasian in white; Indian in black) were assigned on the basis of both the control-region motifs and the coding-region polymorphisms [see Additional file 1] following published criteria (see Materials and Methods). Coding-region markers are reported as mutated nucleotide positions according to the rCRS [17] Mutations are transitions unless a base change is explicitly indicated. The 9-bp polymorphism: deletion = del; insertion = ins. Haplogroups with an asterix (*) include samples negative for the examined sub-groups.

MtDNAs not ascribable to any known or well-defined haplogroup/subhaplogroup were completely sequenced according to Torroni et al. [18]. Overall, 34 novel complete sequences were produced in the course of this study. The assignment of sequences to specific haplogroups was performed as reported in Figure 2, according to the most-recent classifications of Eurasian haplogroups and sub-haplogroups [16,19-31].

Phylogenetic trees were constructed manually and validated by the Network 4.500 program software. Coalescence times for mtDNA haplogroups were calculated by the rho (ρ) statistic according to the mutation-rate estimation of Mishmar et al. [32].

### Y-chromosome analyses

**Figure 3:**
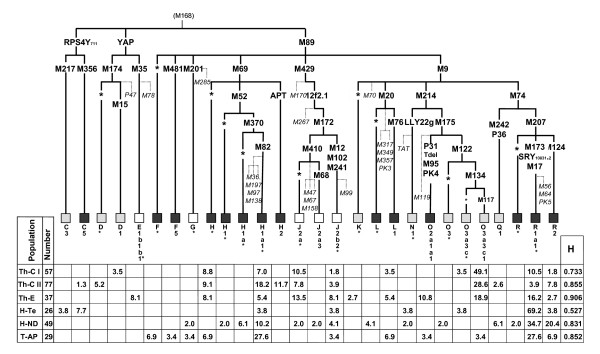
**Phylogeny and frequencies (%) of Y-chromosome haplogroups in the populations studied**. Haplogroups: East Asian in grey; West Eurasian in white; Indian in black. The nomenclature and the hierarchical order of the mutations are according to the Y-Chromosome Consortium [62,67,73], updated with more recent markers: M429 [38]; M481 and P31 T-del (present study). The nomenclature of haplogroup H differs from that presented by Karafet et al. [73], in that all of our M82 samples were also M370 positive. H: intra-population haplogroup diversity, according to Nei [41]. In italics: markers not found. In parentheses: markers inferred. Haplogroups with an asterix* include samples negative for the examined sub-groups.

Y-chromosome haplogroups were defined by the hierarchical order analysis of the 67 MSY bi-allelic markers reported in Figure 3. The YAP, 12f2.1, LLY22g, PK3, PK4, P47 and M429 polymorphisms were analyzed according to Hammer and Horai [33], Rosser et al. [34], Zerjal et al. [35], Mohyuddin et al. [36], Gayden et al. [37] and Underhill et al. [38]. All other mutations were detected by PCR/DHPLC, according to Underhill et al. [39] and, when necessary, results were verified by sequencing fragments of interest.

Twelve STR loci (DYS19, YCAIIa/b, DYS388, DYS389I/II, DYS390, DYS391, DYS392, DYS393, DYS439 and DYS460) were also analysed in the majority of the samples using two multiplex reactions according to  information and by using ABI PRISM^® ^377 DNA Sequencer, internal size standard and GeneScan fragment analysis software.

The age of microsatellite variation within haplogroups was evaluated in samples of five or more subjects according to Sengupta et al. [15] using the mutation rate of 0.00069 per locus per 25 years [40]. Haplogroup heterogeneity (H) was computed using Nei's standard method [41]. Principal Component (PC) analysis was performed on the mtDNA and Y-chromosome haplogroup frequencies using Excel software implemented by Xlstat.

### Web Resource

Accession numbers and the URL for data presented herein are as follows:

GenBank,  for mtDNA complete sequences [GenBank: FJ770939–FJ770973]).

Network software: www.fluxus-engineer-ing.com

STR information: 

## Results

### mtDNA

**Figure 4:**
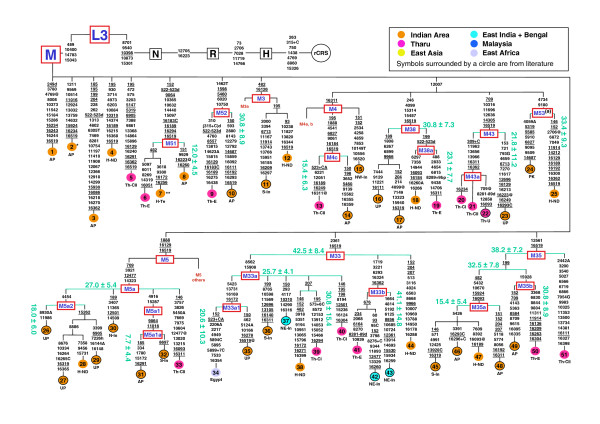
**Phylogenetic tree of 51 mtDNA sequences**. Mutations are scored relative to the rCRS [17]For the tree construction, the length variation in the poly-C stretch at np 16193 was not used, while the variation at np 309 is indicated only when phylogenetically relevant. Mutations are shown on the branches. They are transitions unless a base change is explicitly indicated; insertions are suffixed with a plus sign (+) and the inserted nucleotide(s), and deletions are characterized by "d"; back mutations are highlighted by "@"; recurrent mutations are underlined. Dating is reported in kilo years. Some sequences are incomplete: * from 3694 to 3738 nps; ** from 8353 to 8472 nps. For the ethnic/geographic origins of the samples, see Additional file 2. Population codes: Th-CI and Th-CII: Central Tharus; Th-E: Eastern Tharus; H-ND: Hindus from New Delhi; H-Te: Hindus from Terai; AP: Andhra Pradesh; UP: Uttar Pradesh; N-In, NE-In, S-In: North, North-East, South Indians, respectively; PK: Pakistan. Symbols surrounded by a circle are from literature. (a) Nomenclature different from that (M45) reported in the “mtDNA tree Build 5 (8 Jul 2009)” ().

**Figure 5:**
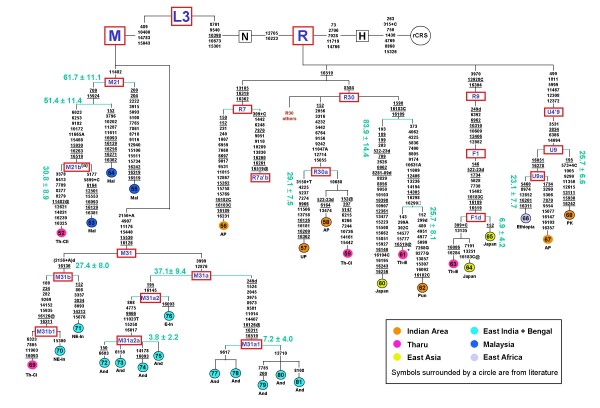
**Phylogenetic tree of 30 mtDNA sequences**. Mutations are scored relative to the rCRS [17]For the tree construction, the length variation in the poly-C stretch at np 16193 was not used, while the variation at np 309 is indicated only when phylogenetically relevant. Mutations are shown on the branches. They are transitions unless a base change is explicitly indicated; insertions are suffixed with a plus sign (+) and the inserted nucleotide(s), and deletions are characterized by “d”; back mutations are highlighted by “@”; recurrent mutations (considered in the global phylogeny of the 81 mtDNAs) are underlined. Dating is reported in kilo years.  * Sequence incomplete from 411 to 628 and from 16189 to 16290 nps. For the ethnic/geographic origins of the samples, see Additional file 2. Population codes: Th-CI and Th-CII: Central Tharus; Th-E: Eastern Tharus; AP: Andhra Pradesh; UP: Uttar Pradesh; Pun: Punjab; NE-In: North-East Indians; E-In: East Indians; PK: Pakistan; Mal: Malaysia; And: Andaman Islands. Symbols surrounded by a circle are from literature.   (a) Nomenclature different from that (M13b) reported in the “mtDNA tree Build 5 (8 Jul 2009)” ().

The mtDNA haplogroups of the examined populations, together with their frequencies, are illustrated in the phylogeny of Figure 2. All M* mtDNAs were sequenced, and only five (1–5 in Figures 4 and 5) did not cluster with other complete sequences. These are reported together as "M others" in Figure 2. The control-region motifs are given in Additional file 1.

Super-haplogroups M (55.7%) and, to a lesser extent, R (39.3%) are the most represented in the dataset. The M lineages were predominant (>50%) in all populations with highest values in the Tharu and Andhra Pradesh samples (75–88% and 76%, respectively). By contrast, the R lineages were present at higher frequencies among Hindus (43.7%) than among the Tharu and the Andhra Pradesh tribals (19.1% and 24.1%) with a few overlaps in the haplogroup distribution. The N(xR) lineages were observed only in three Hindus (4.9%).

The 9-bp polymorphism was found exclusively in the Tharus, associated with three different haplogroups: the deletion (6.4%) with haplogroups B5a (eight subjects) and M33 (three subjects), and the insertion (one subject – 0.6%) with haplogroup M38 (Figures 2 &4).

**Figure 6:**
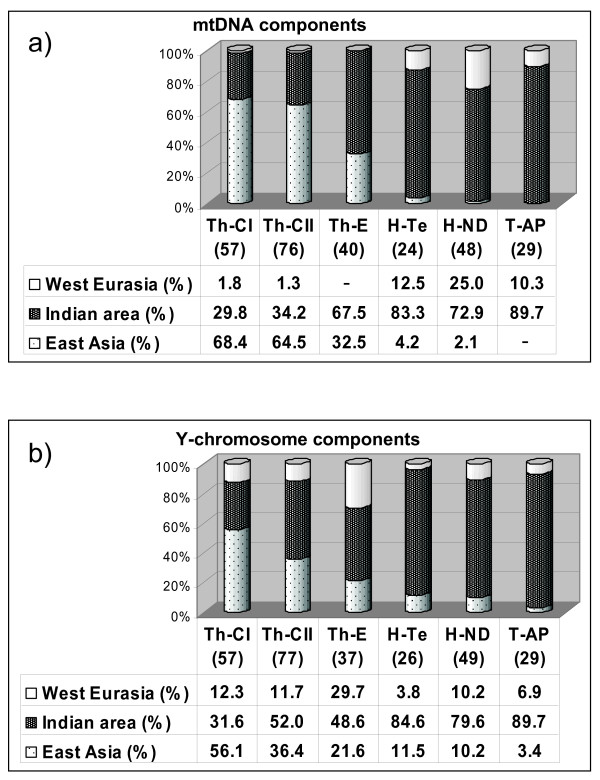
**Histograms of the mtDNA (a) and Y-chromosome (b) components observed in the populations studied**. Sample sizes are in parentheses.

Based on their known or supposed origin [11,20,42-45] it is possible to identify among these haplogroups three main components – East Asian, West Eurasian and Indian – that show a very skewed distribution (Figure 6a).

### The East Asian component

This is represented by nine M mtDNAs belonging to Hgs C, D, G, M9, M21 and Z, and four R mtDNAs belonging to Hgs B5a, and F1. This component, which amounts to about 65% in the two groups of Central Tharus and 33% in the Eastern Tharus, was not observed in the tribals of Andhra Pradesh, and was seen only in two Hindus, one (from Terai) as D4* and the other (from New Delhi) as G*. These two haplogroups, together with the M9a, are among the most frequent in the Tharus, especially group G that includes the G* and G2a, and accounts for 20.8% of the total sample, and for 26.3% of the Th-CI. Interestingly, on the basis of the sequence information of the mtDNA control region (16223, 16274, 16362), the Indian G* appears different from all the other G* haplogroups examined [see Additional file 1], and could belong to haplogroup G3 [29,46]. Haplogroup M21, previously described in Malaysia where it is present with different sub-clades [24,47] has been observed in two Central Tharus, thus establishing a deep correlation with the people of that area. Haplogroup B5a is present in all Tharus, with the highest frequency (8.8%) in the Th-CI group. All these share the 9-bp deletion and the HVS-I motif 16140-16189-16266A, which corresponds to the Nicobar Island B5a1 [25] and is closely related to the Chinese B5a [48,49]. Haplogroup F1 is also present among Tharus as F1*, F1c and F1d. In particular, the subhaplogroup F1c, whose high frequency was reported in Tibeto-Burman tribes of Thailand [50], China [51] and India [52], is found in all Tharu groups.

### The West Eurasian component

This component comprises the N haplogroups I and W, and the R haplogroups R0, H, T2 and U (xU2a,b,c) and is almost absent in the Tharus (only one H and one T2 mtDNAs from Chitwan). In contrast, it reaches a high frequency (25.0%) in New Delhi, where most of the haplogroups of this component are found, and is also common in Indians from Terai (12.5%) and Andhra Pradesh (10.3%). However, in spite of the similar frequencies, the two latter populations are remarkably different in their composition: Hgs I, U1 and T2 characterize the Terai Hindus, whereas Hgs U2e, U5a1 and U9a the Andhra Pradesh tribals.

Among the West Eurasian U sub-clades, particularly interesting are U7 and U9. In the New Delhi sample, U7 shows a frequency (10.4%) that is quite similar to that of Iran (9.4%) and close to its peak (12.3%) in the West Indian state of Gujarat [12]. U9 is a rare haplogroup previously observed in Pakistan [42], Yemen and Ethiopia [23,53]. Interestingly, the U9 mtDNA that we found in Andhra Pradesh, together with an Ethiopian mtDNA, defines the new U9a sub-group (Figure 5), thus confirming the ancient genetic links between East Africa, Southwest Asia and India.

Although the West Eurasian component is probably primarily related to migrations during the Holocene period, the exact source and time of such migrations is difficult to establish [12,45].

### The Indian component

This is the major component of the Indian groups and also of the Eastern Tharus (Figure 6a) and is represented by 36 haplogroups, one third of which are shared between Tharus and Indians and seven are present only among Tharus (Figure 2). Since this component showed a more complex genetic relationship between Tharus and Indians, in addition to the M* samples, other selected mtDNAs were completely sequenced, to obtain a deeper haplogroup phylogeny. The parsimony trees, illustrated in Figures 4 and 5, include 81 sequences, 34 of which are from the present study comprising one exogenous mtDNA (from Egypt), and 47 are from the literature [see Additional file 2] with four exogenous mtDNAs (three from Japan and one from Ethiopia). Ten sequences belonging to Hg M did not enter in any of the previously described haplogroups: five clustered as three new haplogroups **M51**, **M52 **and **M53 **and five resulted as single lineages. The latter five can be used as references for new haplogroup by affiliation of mtDNAs classified only for the control region such as, for example, sequence #3, whose HVS-I motif has been described in one Koya of Andhra Pradesh [11] and one Sudra of Bangladesh [52]. All the other sequences could be assigned to known M and R haplogroups, either as direct basal derivatives or as components of subgroups, contributing to an improved definition of the mtDNA tree and a refinement of age estimates.

### mtDNA phylogeography

The new haplogroups **M51 **and **M52 **were detected in the eastern part of the Indian subcontinent, while **M53 **seems to belong to the West Indian area. As for the new sub-clades of previously described haplogroups, **M4c**, linking one Tharu of Chitwan with one Indian from Andhra Pradesh [30], could be typical of Tribal groups, and **M43a**, is observed at the Indian border with Nepal. Sub-clade **M5a1 **characterizes peoples from North India (New Delhi and Uttar Pradesh [30]), whereas **M5a2 **is present in Southern India [28,30]. Both haplogroups M33 and M35 show many inner branches, but while **M35 **is diffused inside the Indian subcontinent, relating the Tharu groups and the Hindu from New Delhi with populations of South India, **M33 **is also spread elsewhere. Indeed, its sub-clade **M33a **includes one Egyptian mtDNA, thus connecting the Indian subcontinent with North Africa, whereas **M33b**, described in Western Bengalese [30] and in the Indian region of Megalaya [31], has been observed in Eastern Tharus. Therefore, it may represent a clade of the Northeast Indian subcontinent.

Of particular interest is the detection of haplogroups M21 and M31 (two subjects each) among the central Tharus. The Tharu **M21 **sequence (Figure 5) shares nine mutations with one of the three M21 lineages found in all Orang Asli groups of Malaysia [24] and in other groups from Southeast Asia [44], belonging to the sub-group **M21b**. The Tharu **M31 **sequence, together with one Megalaya mtDNA [31], clusters with one West Bengal Rajbhansi [21,27] and defines a sub-group of **M31b**. This subclade, together with M31a2 of the tribal Lodha, Lambadi and Chenchu populations, represents the Indian counterparts of the M31a1 Andaman lineages [27], further supporting a common ancestry of the Indian subcontinent and people of the Bengal Bay islands.

As for the R haplogroups, R7 and R30 are of particular interest. Very informative for the structure and for the age evaluation of haplogroup R7 is the Andhra Pradesh sequence #56 (Figure 5) that defines an extremely deep branch of the R7 in India. This branch shares with the root of the phylogeny of Chaubey et al.[54] only the mutations 13105, 16319 and, in addition, it does not display the 16260 and 16261 mutations characterizing the R7a and R7b branches observed in different R samples from Indian groups [11,52,54-57] and, interestingly, in one R7 Tutsi from Rwanda (unpublished data). Two Tharu mtDNAs, one from Chitwan and one from Eastern Terai, belong to the **R30 **haplogroup. The first is closely related to two Indian sequences, one from Andhra Pradesh and the other from Uttar Pradesh, and contributes to define a sub-clade of the R30a [54]. The second joins a Punjab sequence [54] with a Japanese deep lineage [22] indicating an ancient link between India and Japan. A more recent connection with Japan is, in turn, revealed by the **F1d **haplogroup showing a tight linkage between an Eastern Tharu sequence and two Japanese mtDNAs. Another noteworthy connection with outside areas is evidenced by the **U9 **haplogroup that, being shared by an Ethiopian and an Andhra Pradesh mtDNA, reveals a not recent link between Ethiopia and India.

**Figure 7:**
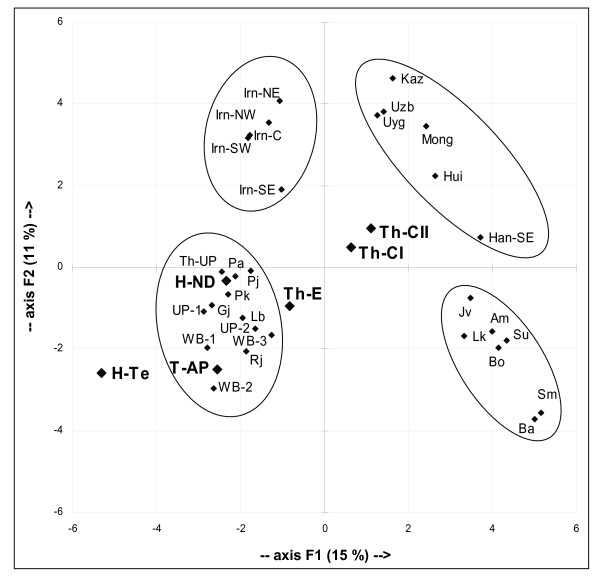
**Principal component analysis of mtDNA haplogroup frequencies**. Comparison samples from Western Eurasia (Iran): Irn-W, Irn-E, Irn-C, Irn-SW, Irn-SE [12]; Indian subcontinent: AP, Andhra Pradesh [55]; WB-1, Castes from Bengal; WB-2, Kurmis from West Bengal; WB-3, Lodhas from West Bengal; Pj, Punjab; Rj, Rajput; Pa, Parsi; Gj, Gujarat; UP-2, Brahmins from Uttar Pradesh [12]; Th-UP, Tharus from Uttar Pradesh [12,56]; UP-1, Uttar Pradesh; Lb, Lobana group [56]; Pk, Karachis [42]; East Asia: Han-SE, Guandong [58]; Uzb, Uzbek; Uyg, Uygur; Kaz, Kazak; Mong, Mongolia; Hui, Xinjiang [59,60]; and Indonesia: Su, Sumatra; Bo, Borneo; Jv, Java; Ba, Bali; Lk, Lombok; Sm, Sumba; Am, Ambon [44]. Data have been normalized to the common level of analysis. On the whole, 26% of the total variance is represented: 15% by the first PC and 11% by the second PC.

Even if the PC analysis of mtDNA haplogroup frequencies observed in the present study compared with those of relevant populations accounts for only about a quarter of the variance, four main clusters are defined: West Eurasian [12], Indian area [12,42,55,56], East Asian [58-60], and Southeast Asian [44] (Figure 7). The first two are well-distinguished from the others by the first PC, which points out a separation between the West and the East Eurasian gene pools; afterwards, the second PC distinguishes West Eurasians from Indians and East Asians from Southeast Asians. Tharu groups are located in the middle of the area among the clusters but, while the central groups are closer to East Asians, Eastern Tharus turned out to be closer to the Indians. Other samples from the border between India and Nepal, such as those from Uttar Pradesh, remain inside the Indian cluster (including the group Th-Up composed of marginal "Hinduized" Tharus [12]. As for Indians, they all group together, in agreement with a deep (Late Pleistocene) common maternal ancestry of caste and tribal populations [11,60], perhaps due to some accepted practices (such as the anuloma) that allow a woman of a lower social level to enter a higher level by marriage [55,61].

### The Y-chromosome

The phylogeny and frequencies of the 28 Y-chromosome haplogroups observed in the present study are shown in Figure 3.

Two new variants are reported. The first, **M481**, defines the new haplogroup F5 and consists of a C→T transition at np 163 within the STS containing the P36 mutation [62]. The second, Tdel, was first noticed in haplogroup **O2-P31 **while typing the P31 marker and was confirmed by sequencing. This is due to a T deletion in the 6T stretch starting at np 127, adjacent to the P31 T to C transition [63]. The T deletion, not found in the other examined Hg O derivatives, is always present in our O2 samples (all tribals; four of the Eastern Tharus and one from Andhra Pradesh). Taking into account that this haplogroup is often recognized through markers different from P31 and that in other studies, where the P31 was examined [64,65], a technique not detecting Tdel was employed, additional DHPLC/sequencing analyses of P31 chromosomes are necessary to evaluate the extent of the contemporary presence of the two mutations. It is worth noting that these samples were also all positive for the PK4 marker recently observed in four Pakistani Pathans [36]. Another variation, consisting of an A to G transition at np 147, was observed in two H-M82 samples while sequencing the M89 marker. This mutation, which was not found either in H-M69* or in H2-APT chromosomes, characterizes the H1 subgroup but, due to the impossibility of typing all the M82 samples, as well as any M370* and M52* Y chromosome, at present, we cannot define the precise phylogenetic position of this novel transition inside the sub-haplogroup.

On the basis of known or supposed haplogroup origin [11,14,15,36,56,62,64-72], three main components (East Asian, West Eurasian and Indian) can also be identified for the Y chromosome. The incidence of the various components in each population is depicted in the histograms of figure 6b.

The **East Asian component **made up by haplogroups C(xC5), D, N, O3, Q, and K*, and mainly represented by Hg O3, is, on the whole, much more frequent among Tharus (39.8%) than among Indians (7.7%). The high Tharu frequency, mostly accounted for by the subgroup **O3-M117 **(83.8%), shows a wide range in the three groups with significant differences between Th-CI *vs *both Th-CII (P < 0.02) and Th-E (P = 0.001). Among the less represented East Asian markers of interest is **Hg D **that is very frequent in Tibet, absent in other Nepalese populations [37] but present in six Central Tharus: as D1-M15 in two Th-CI subjects and as D*-M174 in four Th-CII subjects. The latter, by showing the DYS392 -7 repeat allele that characterizes the D3-P47 chromosomes [37], could belong to the recently identified Hg D3* [73]. In addition, two other haplogroups were encountered: K-M9* in a single Eastern Tharus and **Q1-P36 **in two Tharus-CII. Hg Q, which is present in Tibetans, was seen in only one sample from Kathmandu [37]. In Indians, the very scarce East Asian component was represented by three **Hg O3 **(each belonging to a different sub-haplogroup and to a different Indian sample), one **C3-M217 **in Terai (previously observed only in a few Kathmandu and Tibetan samples [37]), two N1-LLY22g*, one in Terai and one in New Delhi and by three **Q1-P36 **in New Delhi. Only three East Asian haplogroups, Q1-P36, O3-M134* and O3-M117, are shared between Tharus and Indians.

The **West Eurasian **component, represented by haplogroups E, G, and J, shows a higher incidence among Tharus (15.9%) than among Indians (7.7%). With the exception of three E3-M35* Eastern Tharus and two **G**-**M201 **(one in New Delhi and the other in Andhra Pradesh), the main part of this component is accounted for by haplogroup **J **(Tharus 14.0%, Indians 5.8%), present only as J2, namely J2-M410* and J2-M241*. Whereas the latter haplogroup is shared by all Indian and Tharu samples, the J2-M410* was found in all Tharus but in only one Hindu of New Delhi, where one sample of its derivative **J2-M68 **was also present. If one considers the total frequency of this component in each sub-group, among Indians the highest value is observed in the Hindus of New Delhi (10%), and, among Tharus, in the group of Eastern Terai (30%). It is noteworthy that the frequency of Eastern Tharus is about three times higher than that of the other two Tharu samples (P ~ 0.03 *vs *Th-CI and 0.02 *vs *Th-CII). This component may reflect several events of gene flow from the Early Holocene to the present, passing through Neolithic farmers.

The **Indian subcontinent **component includes lineages of haplogroups C, F, H, L, O, R and among Indians it ranges from 80% in the New Delhi sample to 85% in Terai, and to 90% in the Andhra Pradesh. Among Tharus, with the exception of an incidence of ~32% in the Th-CI group, it reaches values around 50% in the other two groups. **Hgs H and R **are the most frequent haplogroups of this component. Hg H (Tharus: 25.7% Indians: 18.3%) is represented by five sub-groups: H-M69*, H1-M52*, H1-M370*, H1-M82* and H2-APT. Whereas H-M69* was detected at similar frequencies (mean 8.8%) in all the Tharu sub-groups, and in two Indians of Andhra Pradesh (6.9%), H1-M82* was seen in all Tharus and Indians. By contrast, H1-M52* (2.0%) and H1-M370* (6.1%) were seen only in the New Delhi Hindus, and **H2-APT **(11.7%) only in the Tharus-CII.

**Hg R**, besides a single R* from New Delhi, was detected in all groups as R1a1-M17* and **R2-M124 **with important differences between Tharus (13.5%) and Indians (52.9%), mainly due to R1-M17* (8.8% *vs *41.3%). Within the two populations, significant differences were also observed: the Tharu-CII sample differs from the Eastern one (3.9% *vs *16.2%, P ~ 0.05); the Hindus from Terai (69.2%) appear very distant from both the New Delhi Hindus (34.7%, P < 0.01) and the Andhra Pradesh tribals (27.6%, P ~ 0.005). However, this important difference could be, at least partially, influenced by the genetic background of the sample that in recent times moved from India to Nepal after malaria eradication.

The Indian component can be resolved into the most likely endogenous (local) haplogroups (C5, F*, H, the two new F5-M481 and O2a1a-T*del*), and the inter-regional ones (L, R1 and R2). In the first group we have included the lineage HgO2-P31-T*del *found in the tribals of both Eastern Tharu and AP Indian samples. The T deletion further characterizes the HgO2-M95 clade that is considered a genetic footprint of the earliest Palaeolithic Austro-Asiatic settlers in the Indian subcontinent [14,71,74], and also as an autochthonous Indian Austro-Asiatic population marker [72]. The remaining endogenous haplogroups include haplogroup C5-M356, shared between Indians and Tharus (two in the Terai Hindus and one in the Tharus-CII), haplogroup F-M89* and its new derivative F5-M481, both considered as tribal markers and observed in Andhra Pradesh (10.3%). As for the inter-regional haplogroups L-M20, R1-M17 and R2-M124, they display within India a considerable frequency and haplotype associated high microsatellite variance. However, whereas this observation for the subgroup L1-M76 of L-M20 and for R2-M124 showing lower frequencies outside this region, is considered indicative of a local origin, for R1-M17 the situation is more complex, as well as the position of L-M20*. Actually, the high frequency of the R1-M17 haplogroup found in the Central Eurasian territory, together with its gradient of diffusion that was associated with the Indo-European expansion [74-76], would leave some uncertainty about its geographic origin. However, the high microsatellite variation supports an ancient presence, dated in our samples over 14 ky [see Additional file 3] of the M17 marker in the Indian subcontinent, as suggested by Kivisild et al. [11], and sustained by Sengupta et al. [15] and Thanseem et al. [71], who consider the Indo-European M17 only a contribution to a local Early Holocene pre-existing Indian M17. Thus, it is reasonable to assume that even this inter-regional haplogroup has ancient relationships with the Indian area. Interestingly, the M17 Y-chromosomes of the Indian subcontinent differentiate from those of Central Eurasia in that they are virtually all 49a,f/*Taq*I Ht 11 [77].

As to the rare haplogroup L-M20*, it was present in two individuals of the New Delhi sample. Only one of these Y-chromosomes could be analyzed for the microsatellites and compared in a network with other seven available samples L-M20* of Turkish and Italian origin (unpublished data), showing that it was very distant from the others.

Age estimates of the main haplogroups with some comparative data [15] are reported in Additional file 3. Although age estimates deserve caution, particularly when samples are small and standard errors large, a good general agreement between the two datasets is observed. As for haplogroup H1-M82*, not reported by Sengupta et al. [15], its age is very similar in all groups, with variance (0.093–0.110) lower than that (0.19) previously observed in some Indian groups [11]. Special attention is deserved by haplogroups J2-M410*and R1-M17*, showing variances very different in the various Tharu and Indian sub-groups and the highest values in the Eastern Tharus and tribals of Andhra Pradesh. Interesting is also Hg R2-M124 for which the Tharu total variance rises to 0.271, a value obtained by adding just two samples from the other Tharu groups to six homogeneous Th-CII samples (variance 0.033), thus stressing again the Tharu heterogeneity.

**Figure 8:**
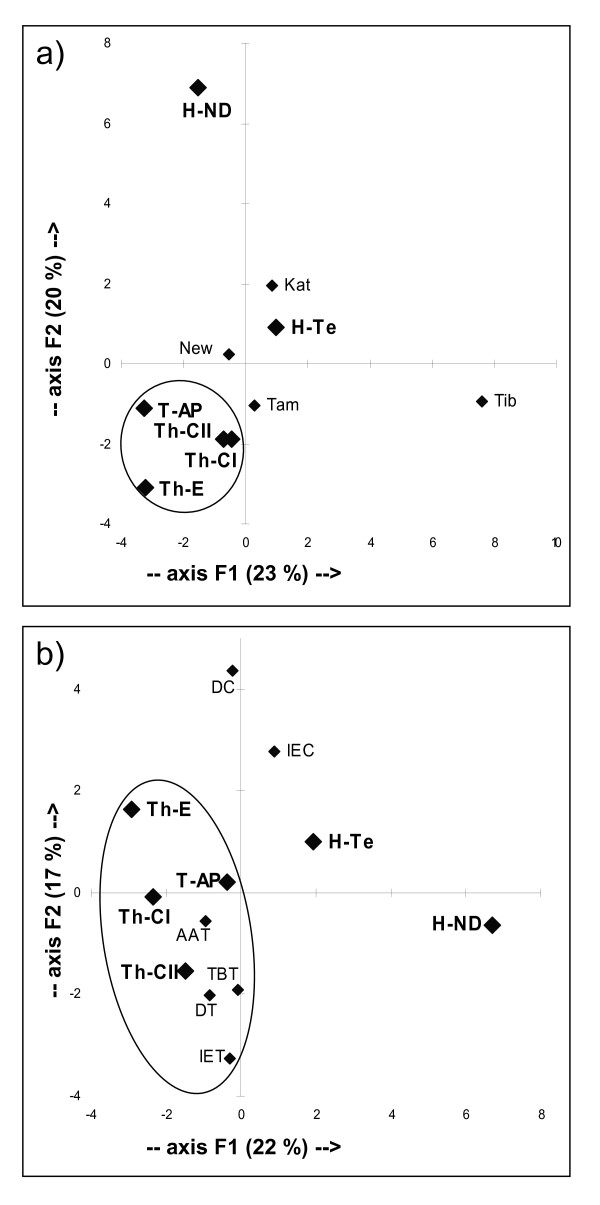
**Principal component analysis of Y-chromosome haplogroup frequencies**. (a) Comparison with Nepalese and Tibetan groups [37]; (b) Comparison with some Indian caste and tribal groups [15] where our data have been normalized to the Sengupta level of resolution. Populations: Kat, Kathmandu; New, Newar; Tam, Tamang; Tib, Tibet; DC Dravidian castes; IEC, Indo-European castes; AAT, Austro-Asiatic tribals; TBT, Tibeto-Burman tribals; DT, Dravidian tribals; IET, Indo-European tribals.

The PC analyses of the haplogroup frequencies, which were performed with the Nepalese and Tibetan data of Gayden et al. [37] and the Indian caste and tribal groups of Sengupta et al. [15], are illustrated in Figure 8a,b. In both plots, a cluster of tribals, including Tharus and the Indians from Andhra Pradesh, is evident and separated from the caste groups. As for the Nepalese populations, all are very distant from Tibetans. Tharus, with the Eastern group always in a peripheral position, cluster together in the same quadrant of the plot, distinct from those occupied by the other three Nepalese groups.

## Discussion

The analysis of mtDNA and Y chromosome polymorphisms in three Tharu samples from Central and Eastern Terai has enlightened the presence of three main components, Oriental, West Eurasian and Indian, that show remarkable quantitative and qualitative differences among the three groups as well as between sexes within the same group.

### The East Asian signature of the Tharus

Like Tibetans and other people of Nepal [37] the greater part of the East Asian influence in the Tharus may be mainly traced back to Tibeto-Burman speakers who entered Northeast India within the last 4.2 ky [78] and likely influenced them through a founder effect. Indeed, **East Asian mtDNA **haplogroups present in the Tharu samples show lower genetic variation: all control-region haplotypes are similar [see Additional file 1] and do not cover the variety found within the Tibeto-Burman populations [79]. In particular, B5a, D4, G2a mtDNAs are present among Tharus, whereas B4, D5 mtDNAs as well as haplogroups A, M7 and R10 were not observed. Signatures of this influence are also seen in the Tharu Y chromosomes that are almost completely represented by haplogroup O3-M117. Interestingly, Tibetan markers not present in the other Nepalese populations [37] are revealed in the Central Tharus by haplogroups D (4.5%) and Q (0.7%) of the Y chromosome.

### The Middle Eastern signature of the Tharus

West Eurasian markers are virtually absent in the mtDNA of Tharus, whereas they are present in their Y chromosomes essentially as J2-M410* and J2-M241*, with a frequency peak (30%) in the eastern sample, where three E-M35 chromosomes were also observed. These latter, all displaying the same microsatellite haplotype, could be attributed to recent gene flow from the Middle East or, as previously reported for the Indian Siddis, from Africa [80,81]. By contrast, both sub-haplogroups of J are indicative of various connections with the Middle East. J-M410, which was associated with the first farmer dispersal in Europe [13,82-84], shows variance values of 0.346 in the Tharus and 0.339 in Indian groups [15]. These values are lower than those (0.467 and 0.479) observed in Anatolia [13,82] and (0.410) in Southeast Europe [83,84] and therefore are compatible with a dispersal of this lineage from somewhere in the Middle East/Asia Minor. The situation of J-M241* is more difficult to interpret. The variance of this lineage shows a value of 0.437 in the Tharus which is higher than that (0.328) obtained from the Indian data of Sengupta et al. [15], thus suggesting a pre-Neolithic presence of J-M241* in the Indian subcontinent.

### The Indian background

A great majority of the Tharu mtDNA and Y-chromosome gene pools is represented by lineages shared or derived from Indian haplogroups. In particular, Tharus share with Indians ancient mtDNA haplogroups (see for example, the M clades M31, M33, M35, M38, the new M52 and also the R30, almost all dated ~30 ky) and Y-chromosome haplogroups (such as H-M69, O2-P31Tdel, R1-M17* and R2-M124) that, in the isolated malaria-resistant Tharus of Terai, could be retained. Therefore, Tharus might have been structured *in situ *by major demographic episodes of the past, and then by relatively minor gene flows due to subsequent migrations.

### Tharu gene pool: a reservoir of variation generated by local differentiations and by traces of different migratory routes

The remarkable qualitative heterogeneity of the three components and of the age of their haplogroups in the total populations and in their sub-groups [see Figures 4 and 5 and Additional file 3] makes it possible to set them in a temporal background and to identify links between the various populations of the Indian subcontinent, as well as with populations outside this area.

**Figure 9:**
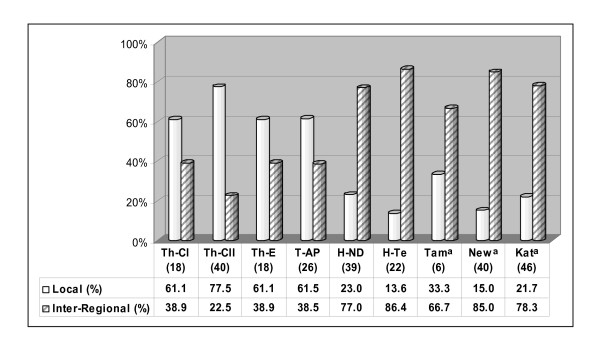
Histograms of the Indian local (Hgs: C5, F, H, L1, O2a1a1) and inter-regional (Hgs: L* and R) components observed in the populations of the present study compared with other Nepalese groups. Sample sizes are in parentheses. (a) Gayden et al. [37]

Of particular interest is the link emerging between Tharus and tribals from Andhra Pradesh, as well illustrated by the Y-chromosome PCA plots (Figure 8) and by the high prevalence in these two populations of the local Y-chromosome haplogroup component (Figure 9), in comparison to the Hindus and to the other populations of Nepal [37] where the inter-regional component is clearly predominant. This further supports a deep common ancestry between Tharus and Indians, probably due to the legacy of the first settlers who arrived from the Indian coasts during the out-of-Africa dispersal. Subsequently, the high level of consanguinity inside numerous social boundaries, along with the influences of evolutionary forces such as long-term isolation, could be responsible for the development of local genetic variants stemming out from the same founders, as seen for mtDNA haplogroups M43, M51, M52, R30a in figures 4 and 5.

Useful in further elucidating and deepening these processes has been the complete sequencing of informative mtDNAs, especially belonging to haplogroup M.

The links between the Central Tharus and the Andaman Islanders through Northeast India (Hg M31), between the Eastern Tharus and Japan (Hg R30) and between Central Tharus and Malaysia (Hg M21), are ancient. However, whereas our results are in agreement with an Indian ancestor for haplogroup M31 [27], they are not informative about the origin of haplogroup M21 (observed in two Tharus-CII), given its Southeast Asian frequency and variation [44]. Haplogroup R30 could represent a relic of the postulated out-of-Africa South Coastal Route [24], whereas M33, together with U9a, indicate ancient links of India with North and East Africa. These events of gene flow, however, according to the divergence times (20.6 + 10.3 and 23.1 + 7.7 ky, respectively), would have occurred more recently than those previously described and dated to about 40–45 ky [43].

### Sex-specific influences

Clear sex-biased frequencies emerged from these analyses. This is particularly evident for the East Asian contribution that shows a decreasing trend from Central to Eastern Tharus and is more strongly represented in the mtDNA than in the Y-chromosome data set. By contrast, the West Eurasian contribution, extremely scarce and even absent in the Tharu mtDNA, accounts from 12% to 30% of the Y-chromosome data set. As for the Indian component, it is well represented in all groups, with the highest frequencies in the Eastern Tharu mtDNA and in the Y chromosomes of Tharu-CII.

Apart from genetic drift, these sex-specific influences can be ascribed to all those human movements with different male/female composition. Thus, whereas the first human dispersals involved both males and females, more recent immigrations, involving mainly men [85], gradually diluted the ancient local Y-chromosome pool. A clear example of a recent sex-biased influence emerged in the comparison between lower and the northern upper casts, the latter receiving in the last few thousand years, a Indo-European male genetic input from the North [86,87]. Thus, the differentiation between tribal and non tribal groups is evident for the Y chromosome (Figure 8) whereas a major similarity characterizes the two groups for mitochondrial DNA (Figures 7).

### Comparison with other Nepalese populations

By considering the Nepalese populations examined by Gayden et al. [37], apart from the homogeneous Tamang sample that displays almost exclusively the East Asian haplogroup O3-M134, the Newar and Kathmandu groups, like Tharus, show an important Indian component. However, whereas in the first two, the inter-regional haplogroups are most represented, in the Tharus the local ones are prevalent (Figure 9). Both quantitative and qualitative differences emerge from the East Asian component: on the whole it is most frequent and heterogeneous among Tharus, especially in the Chitwan groups which, in addition to the frequent Hg-O3-M117, show the Hgs D and Q, reflecting a Tibetan influence. The West Eurasian component, virtually absent in the Tibetan sample, is represented in Newar and Kathmandu groups with frequencies of 7.6% and 10.4%, respectively. It is interesting to note however, that the Newar sample in addition shows a substantial presence (10.6%) of the R1-M269 haplogroup not found in all the other examined populations.

## Conclusion

The analyses carried out on the mtDNA and Y chromosome of the Tharus, one of the oldest and the largest indigenous people of Terai, have shown a complex genetic structure within which are identifiable: **i) **a deep common ancestry between Tharus and Indians, not previously reported, more evident for mtDNA but also revealed by the prevalence of the local Indian Y-chromosome subcomponent, as in the tribals of Andhra Pradesh; **ii) **a significant East Asian genetic contribution both in the male and female gene pool; **iii) **a western heritage, clearly evident for the Y-chromosome; **iv) **a remarkable heterogeneity of the Tharu population (with the Eastern Tharus more dissimilar to the others) ascribable both to various exogenous influences and to subgroup specific lineages stemming from a shared genetic background with Indians.

Particularly informative has been the complete mtDNA sequencing that further supports a deep differentiation of mtDNA haplogroups in the Indian subcontinent, indicating that some branches are geographically or socially specific, while others are widespread. The improvement in the mtDNA phylogeny has also allowed the identification of ancient relationships between Tharus, not only with the Indian subcontinent area, including Pakistan, but also with the Andaman Islands, Malaysia, and Japan, as well as between India and North and East Africa. The new sequence data also allow a better definition of the genetic relationships among Indian populations at the microgeographic level. Indeed many control-region data from the literature, if compared to the mtDNA sequences of the present study can now be classified into known haplogroups.

Moreover, the importance of genetic isolates in revealing variants not easily detectable in the general population has clearly emerged.

## Authors' contributions

SAS-B and OS, designed the research; GM collected samples; SF, MP, RM, generated the mtDNA data; SF, VB, RM generated the Y-chromosomal data; OS, SF, MP, VB, and AA carried out the statistical analyses. SAS-B, OS and AT wrote the paper. All authors discussed the results and commented the manuscript.

## Supplementary Material

Additional file 1 **MtDNA control and coding regions information of the population samples examined**. The data provide the markers examined in the subjects of the present study. Click here for file

Additional file 2 **Additional file 2**. **Origin of the Figure **4**mtDNA complete-sequences**. The data provide information on the completely sequenced mtDNA molecules of Figures 4 and 5. Click here for file

Additional file 3 **Ages of the main Y-chromosome haplogroups in the samples of the present study together with relevant comparative data from Sengupta et al**. [15]. Age estimates of the main Y-chromosome haplogroups in the different population samples of the present study compared with those reported by Sengupta et al. [15]. Click here for file
