# Supplementary material for: Mitochondrial and Y-chromosome diversity of the Tharus (Nepal): a reservoir of genetic variation
Source: BMC Evol Biol. 2009 Jul 2;9:154. doi: 10.1186/1471-2148-9-154 (PMC2720951; doi:10.1186/1471-2148-9-154)
Supplement: Additional file 2 — Origin of the Figure 4mtDNA complete-sequences. The data provide information on the completely sequenced mtDNA molecules of Figures 4 and 5. [file 1471-2148-9-154-S2.doc]

**Additional file 2.** Origin of the Figure 4 mtDNA complete-sequences.

| **ID** | **Sample Code** | **Sample**  **Haplogroup** | **Sample Origin** | **Sample**  **Location** | **Sequence Reference** |
| --- | --- | --- | --- | --- | --- |
| 1 | AP-21 | M | Indian Andhra Pradesh | South India | This study |
| 2 | AP-70 | M | Indian Andhra Pradesh | South India | This study |
| 3 | AP-88 | M | Indian Andhra Pradesh | South India | This study |
| 4 | HND-8887 | M | Hindu New Delhi | North India | This study |
| 5 | ThC II-71 | M | Tharu Chitwan district | Terai-Nepal | This study |
| 6 | ThE-14 | M51 | Tharu Morang district | Terai-Nepal | This study |
| 7 | HTe-64 | M51 | Hindu Terai | Terai-Nepal | This study |
| 8 | AP-6 | M51 | Indian Andhra Pradesh | South India | This study |
| 9 | ThE-36 | M52 | Tharu Morang district | Terai-Nepal | This study |
| 10 | R58 | M52 | Reddy Andhra Pradesh | South India | Sun et al. [30] |
| 11 | P12 | M3 | Paniya Kerala | South India | Thangaraj et al. [26] |
| 12 | HND-8879 | M3 | Hindu New Delhi | North India | This study |
| 13 | ThC II-3 | M4c | Tharu Chitwan district | Terai-Nepal | This study |
| 14 | R81 | M4c | Reddy Andhra Pradesh | South India | Sun et al. [30] |
| 15 | A8.JS | M4 | Tadvi Gujarat | Northwest India | Thangaraj et al. [26] |
| 16 | A24 | M38 | Bhargava Uttar Pradesh | North India | Sun et al. [30] |
| 17 | T72 | M38 | Thogataveera Andhra Pradesh | South India | Sun et al. [30] |
| 18 | HND-8913 | M38 | Hindu New Delhi | North India | This study |
| 19 | ThE-26 | M38 | Tharu Morang district | Terai-Nepal | This study |
| 20 | ThC I-497 | M43a | Tharu Chitwan district | Terai-Nepal | This study |
| 21 | ThC II-50 | M43a | Tharu Chitwan district | Terai-Nepal | This study |
| 22 | T6 | M43a | Tharu Uttaranchal | North India | Thangaraj et al. [26] |
| 23 | B177 | M43 | Chaturvedi Uttar Pradesh | North India | Sun et al. [30] |
| 24 | AS47 | M53 | Sindhi Pakistan | Pakistan | Kivisild et al. [28]^(a)^ |
| 25 | HND-8904 | M53 | Hindu New Delhi | North India | This study |
| 26 | B47/66 | M5a1 | Chaturvedi Uttar Pradesh | North India | Sun et al. [30] |
| 27 | C8 | M5a1 | Brahmin Uttar Pradesh | North India | Sun et al. [30] |
| 28 | HND-8876 | M5a1 | Hindu New Delhi | North India | This study |
| 29 | C48 | M5a1 | Brahmin Uttar Pradesh | North India | Sun et al. [30] |
| 30 | OR89 | M5a | Gadaba Orissa | Northeast India | Thangaraj et al. [26] |
| 31 | R61 | M5a2 | Reddy Andhra Pradesh | South India | Sun et al. [30] |
| 32 | AS32 | M5a2 | Indian Tamil | South India | Kivisild et al. [28]^(a)^ |
| 33 | ThC II-117 | M5a | Tharu Chitwan district | Terai-Nepal | This study |
| 34 | SI52 | M33a1 | Berber Siwa | Egypt | This study |
| 35 | C182 | M33a1 | Brahmin Uttar Pradesh | North India | Sun et al. [30] |
| 36 | A21.JS | M33a | Phardan | South India | Thangaraj et al. [26] |
| 37 | Ga11 | M33a | Meghalaya | Northeast India | Reddy et al. [31] |
| 38 | HND-8882 | M33a | Hindu New Delhi | North India | This study |
| 39 | ThC I-431 | M33a | Tharu Chitwan district | Terai-Nepal | This study |
| 40 | ThC I-424 | M33a | Tharu Chitwan district | Terai-Nepal | This study |
| 41 | ThE-34 | M33b | Tharu Morang district | Terai-Nepal | This study |
| 42 | SW23 | M33b | Rajbhansi West Bengal | Northeast India | Sun et al. [30] |
| 43 | Pn14 | M33b | Meghalaya | Northeast India | Reddy et al. [31] |
| 44 | HND-8877 | M33 | Hindu New Delhi | North India | This study |
| 45 | M306 | M35a | - | South India | Ingman and Gyllensten [19] |
| 46 | R134 | M35a | Reddy Andhra Pradesh | South India | Sun et al. [30] |
| 47 | HND-8874 | M35a | Hindu New Delhi | North India | This study |
| 48 | R114 | M35a | Reddy Andhra Pradesh | South India | Sun et al. [30] |
| 49 | T17 | M35b | Thogataveera Andhra Pradesh | South India | Sun et al. [30] |
| 50 | ThE-31 | M35b | Tharu Morang district | Terai-Nepal | This study |
| 51 | ThC II-45 | M35 | Tharu Chitwan district | Terai-Nepal | This study |
| 52 | ThC II-39 | M21b | Tharu Chitwan district | Terai-Nepal | This study |
| 53 | #6 | M21b | Jahai Orang-Asli Semang | Malaysia | Macaulay et al. [24] |
| 54 | #5 | M21 | Batek Orang-Asli Semang | Malaysia | Macaulay et al. [24] |
| 55 | #10 | M21 | Semelai Orang-Asli Aboriginal Malay | Malaysia | Macaulay et al. [24] |
| 56 | AP-15 | R7 | Indian Andhra Pradesh | South India | This study |
| 57 | C60 | R30a | Brahmin | - | Palanichamy et al. [20] |
| 58 | T124 | R30a | Thogataveera Andhra Pradesh | South India | Palanichamy et al. [20] |
| 59 | ThC I-451 | R30a | Tharu Chitwan district | Terai-Nepal | This study |
| 60 | ONsq042 | R30 | - | Japan | Tanaka et al. [22] |
| 61 | ThE-16 | R30 | Tharu Morang district | Terai-Nepal | This study |
| 62 | Pun47 | R30 | Punjab | Punjab | Chaubey et al. [54] |
| 63 | ThE-32 | F1d | Tharu Morang district | Terai-Nepal | This study |
| 64 | TCsq069 | F1d | - | Japan | Tanaka et al. [22] |
| 65 | NDsq190 | F1d | - | Japan | Tanaka et al. [22] |
| 66 | #11 | U9a | - | Ethiopia | Achilli et al. [23] |
| 67 | AP-66 | U9a | Indian Andhra Pradesh | South India | This study |
| 68 | #12 | U9b | - | Pakistan | Achilli et al. [23] |
| 69 | ThC I-401 | M31b1 | Tharu Chitwan district | Terai-Nepal | This study |
| 70 | Bh23 | M31b1 | Meghalaya | Northeast India | Reddy et al. [31] |
| 71 | SW1 | M31b | Rajbhansi West Bengal | Northeast India | Endicott et al. [27]; Palanichamy et al. [21] |
| 72 | A12.JS | M31a | Andaman Islands | Southeast Asia | Thangaraj et al. [26] |
| 73 | A14.JS | M31a | Andaman Islands | Southeast Asia | Thangaraj et al. [26] |
| 74 | A15.JS | M31a | Andaman Islands | Southeast Asia | Thangaraj et al. [26] |
| 75 | A13.JS | M31a | Andaman Islands | Southeast Asia | Thangaraj et al. [26] |
| 76 | Lo/Ch/La | M31a | Lodha, Chenchu, Lambadi | East India | Endicott et al. [27] |
| 77 | GA11 | M31a | Andaman Islands | Southeast Asia | Thangaraj et al. [25]; Palanichamy et al. [21] |
| 78 | GA15 | M31a | Andaman Islands | Southeast Asia | Thangaraj et al. [25]; Palanichamy et al. [21] |
| 79 | O23 | M31a | Andaman Islands | Southeast Asia | Thangaraj et al. [25]; Palanichamy et al. [21] |
| 80 | A10.JS | M31a | Andaman Islands | Southeast Asia | Thangaraj et al. [26] |
| 81 | A11.JS | M31a | Andaman Islands | Southeast Asia | Thangaraj et al. [26] |

Note:

1. available only from np 435 to np 16093.
